# Vaccines targeting the neovasculature of tumors

**DOI:** 10.1186/2045-824X-3-7

**Published:** 2011-03-08

**Authors:** Agata Matejuk, Qixin Leng, Szu-Ting Chou, Archibald J Mixson

**Affiliations:** 1 Department of Pathology, University of Maryland Baltimore, MSTF Building, 10 South Pine Street, Baltimore, MD 21201, USA; 2 Department of Plastic Surgery, Microsurgery Laboratory, Cleveland Clinic Foundation, 9500 Euclid Avenue, Cleveland Ohio 44195, USA

## Abstract

Angiogenesis has a critical role in physiologic and disease processes. For the growth of tumors, angiogenesis must occur to carry sufficient nutrients to the tumor. In addition to growth, development of new blood vessels is necessary for invasion and metastases of the tumor. A number of strategies have been developed to inhibit tumor angiogenesis and further understanding of the interplay between tumors and angiogenesis should allow new approaches and advances in angiogenic therapy. One such promising angiogenic approach is to target and inhibit angiogenesis with vaccines. This review will discuss recent advances and future prospects in vaccines targeting aberrant angiogenesis of tumors. The strategies utilized by investigators have included whole endothelial cell vaccines as well as vaccines with defined targets on endothelial cells and pericytes of the developing tumor endothelium. To date, several promising anti-angiogenic vaccine strategies have demonstrated marked inhibition of tumor growth in pre-clinical trials with some showing no observed interference with physiologic angiogenic processes such as wound healing and fertility.

## Introduction

Cancer mortality is related to the spread of neoplastic cells to distant loci where the cells, supported by existing blood vessels and angiogenesis, proliferate and give rise to secondary tumors. Tumor angiogenesis is up-regulated by a number of conditions including hypoxia, hypoglycemia, mechanical disruption, and genetic and inflammatory alterations [1] that lead to activation of growth factors and pro-angiogenic genes [2,3]. The stringent regulation of angiogenesis in normal tissues is often lacking in tumor angiogenesis, resulting in immature and leaky tumor vessels. Furthermore, compared to the tissue-vessel distribution in normal tissue, there is an uneven distribution of vessels within tumors, leading to tumor hypoxia and inefficient transport of chemotherapeutic drugs. In contrast to normal endothelial cells, in which the vast majority are quiescent, tumor endothelial cells actively proliferate, driven by hypoxia and increased levels of angiogenic factors and their cognate receptors. These differences between quiescent and angiogenic endothelial cells resulted in the first clinical anti-angiogenesis trial on human cancer two decades ago. There are now several anti-angiogenic therapies that have received FDA approval including sunitinib, sorafenib, and bevacizumab; and with more than 40 anti-angiogenic drugs in clinical trials [4], further advances are anticipated [5-11].

Differences among tumor endothelial cells and non-malignant endothelial cells may not only be quantitative but in some instances may also be qualitative. With serial analysis of gene expression, investigators compared gene expression from endothelial cells isolated from normal or malignant tissue, and found that several transcripts (e.g., CD276) were specifically elevated in the tumor endothelium [12,13]. Although most receptors/proteins that are increased in the tumor endothelium are also up-regulated in physiologic angiogenic processes, CD276 is not increased in the vessels of wounds or the corpus luteum [13]. Nevertheless, CD276 is not completely specific for the tumor endothelium because its expression may be induced by cytokines on the cell surfaces of B cells, T cells, and dendritic cells. There are also many proteins/receptors in tumor endothelial cells that are overexpressed (such as VEGFR2 and survivin) compared to expression in quiescent endothelial cells. Proteins differentially expressed on tumor endothelial cells or the supporting matrix are attractive targets for vaccine strategies, with the goal of breaking tolerance to self-antigens.

Targeting the tumor vasculature with vaccines as well as with other immunotherapies may have several potential advantages over targeting tumor cells. First, tumor endothelial cells are more accessible to the immune system than are tumor cells at a distance from the vessels. Second, endothelial cells of the tumor are usually more stable genetically than tumor cells, thereby reducing the risk of resistance developing to immunotherapies [14,15]. Chromosomal abnormalities, however, have been identified in endothelial cells of solid tumors [16,17], and in glioblastomas, the tumor cells and its endothelium are derived from common cancer stem-like cells [18,19]. Third, down-regulation of MHC I in tumor cells occurs less frequently in tumor endothelial cells, thereby leading to a more potent CD8+-mediated response. Fourth, since inhibition of a single endothelial cell can inhibit up to 100 tumor cells [20,21], immunotherapies directed toward tumor endothelial cells have the potential of an amplifying inhibitory effect.

As a result of these putative advantages and differentially expressed proteins in the tumor endothelium, a number of immunotherapeutic strategies have targeted angiogenesis, including monoclonal antibodies, vaccinations, and adjuvant co-stimulatory therapies [1]. The most successful of these approaches, thus far, has been passive immunotherapy by utilizing monoclonal antibodies. In 2004, the monoclonal antibody bevacizumab which targets angiogenesis through VEGF received approval for treatment of colorectal cancer [22]. Bevacizumab has also shown efficacy against other cancers including lung, renal, and breast cancers [23,24]. It is likely that the success and ability of bevacizumab to selectively target tumor endothelial cells has provided impetus to development of other forms of angiogenic immunotherapies. Several promising preclinical studies of tumor endothelial vaccines have led to clinical trials that are primarily in phase I. In the burgeoning field of tumor immunotherapies, we will focus on tumor vaccines that have a major anti-angiogenic component.

## Delivery Systems of Tumor Endothelial Vaccines

As this review will highlight, there are many promising tumor endothelial vaccines with demonstrated efficacy in various animal models. These vaccines have been delivered by different approaches/vectors, including direct inoculation of peptides or "naked DNA", gene gun with gold particles, intradermal electroporation, tumor or dendritic cell-based vectors, and attenuated live bacteria vectors. The particular delivery system for anti-angiogenic vaccine therapy is selected at least in part based on whether immunizations are comprised of peptides/proteins, DNA, or RNA. For peptide delivery systems, the peptide can be inoculated directly into the animal model along with an adjuvant, or dendritic cells can be pulsed with the peptides before their inoculation. For gene therapy vaccine approaches, recombinant DNA may be delivered alone ("naked DNA"), by non-viral and viral carriers, or by eukaryotic and prokaryotic cells. Although delivery systems for vaccines targeting tumor endothelial cells generally mirror those targeting tumor cells [25-29], there are exceptions such as the infrequent use of viruses with tumor-endothelial vaccines. Nevertheless, we see no contraindication to using modified herpes simplex or vaccinia viruses to augment the immune response of endothelial vaccines as in tumor cell vaccines.

To date, plasmids encoding angiogenic self-antigens are the most common forms of nucleic acid to demonstrate an anti-angiogenic effect in mouse models. Moreover, bacteria have been the most frequently used delivery system for plasmid-based vaccines (see reviews of [30,31]). Of the 32 vaccines with specific targets covered in this review, bacteria were the primary delivery vector in 11 studies, whereas direct inoculation of "naked" plasmid DNA was the primary delivery system in 6 studies (see Table 1). Several animal studies have demonstrated that orally administered bacteria-based vectors with attenuated, nonreplicating strains of *Listeria *or *Salmonella *have the potential to prevent and treat cancer through inhibition of angiogenesis [32-35]. Although safety concerns are a factor in considering these bacterial delivery systems, it is of note that one *Salmonella enterica *strain has been approved by the FDA for vaccine use [30,36]. Moreover, several bacteria-based vaccines that had marked anti-angiogenic and anti-tumor activity showed little to no autoimmune response, at least in the animal studies. Electroporation is also an appealing approach that has been used with DNA or RNA vaccines that target the tumor endothelium [37,38]. Because of the high number of antigen presenting cells, the skin is a common route of delivery for varied delivery systems including electroporation. The intradermal DNA vaccination approach enables long-term immune protection against tumor angiogenesis and growth. Although electroporation has been used less frequently than direct inoculation of plasmid DNA, it may be more effective. For example, intradermal electroporation of "naked DNA" gave a much stronger anti-angiogenic and anti-tumor immune response to survivin compared to intramuscular DNA injection [37,39,40].

**Table 1:** Different Strategies Utilized With Tumor Endothelial Vaccines

TARGET	FORM OF THE VACCINE	TUMORS	Vector/Route	MECHANISMS	Other Comments	REFERENCES
**Endothelial Cell Targets**						

**VEGFR2**	mVEGFR2-AP fusion protein	Melanoma and lung carcinoma	DC pulsed	Ab, CTL Primarily CD8+	P	Li Y et al., 2002 [45]
	
	Autologous DNA vaccine- full-length mVEGFR2	Melanoma, colon carcinoma, non-small cell lung carcinoma, hepatoma	*S. typhimurium*, oral	CTL	Modest delay in wound healing P, T	Niethammer AG et al., 2002 [59]
	
	Xenogeneic DNA vaccine	Murine melanoma, carcinoma, fibrosarcoma, lyphoma	"Naked" DNA, SC	Ab, CTL, CD4+(Th1)-mediated	Quail VEGFR2 vaccine Increased levels of IgG2a and 2b P, T	Liu, J-Y et al., 2003 [130]
	
	Autologous DNA vaccine- mVEGFR2 fragment	Breast tumor-rat *Her2 *expressing carcinoma; murine p53- deficient breast carcinoma	*L. monocytogenes*, oral	CD8+ mediated Inf-γ Elispot	Encodes listerolysin-VEGFR2 fragment; No effect on wound healing or pregnancy P, T	Seavey MM et al., 2009 [32]
	
	Autologous DNA minigene	Murine breast and colon carcinomas	*S. typhimurium*, oral	CTL	Encodes H-2Kd or H-2Dd restricted peptides P	Luo Y et al., 2007 [63]
	
	Autologous DNA minigene	Murine lung, prostate, and breast cancers	*S. typhimurium*, oral	CTL	Plasmid also encodes HIV-TAT peptide P	Zhou H et al., 2005 [61]
	
	H-2D^b ^-restricted Peptides	Murine colon carcinomas	SC	CTL	adjuvant (GM-CSF, CD40 Ab); T	Dong Y et al., 2006 [62]
	
	HLA-A2 or-A24 restricted hVEGFR2 Peptides	Mouse melanoma and colon carcinomas	ID	CTL	HLA-24 restricted Peptide 169 (RFVPDGNRI) induced human PBMC-CTL lysis of endothelial cells	Wada S et al., 2005 [43]
	
	VEGFR2 Peptide 169 + gemcitabine	Pancreatic cancer (Phase I)	SC	CTL; Reduced Treg cells	Adjuvant (IFA)	Miyazawa M. et al., 2009 [66]
	
	Xenogeneic DNA vaccine	Murine breast and colon carcinoma	Cationic liposomes, IV	Ab, CTL	Human VEGFR2; P, T	Xie K et al., 2009 [65]
	
	Autologous DNA vaccine (VEGFR2 fused with β-defensin 2)	Murine lung and colon cancer	Cationic liposomes, IM	Ab, CTL	Antitumor and anti-angiogenic synergy between VEGFR2 and β-defensin-2; P, T	Wang YS et al., 2007 [64]
	
	Autologous DNA vaccine- Extracellular Domain	Murine Lung	*S. typhimurium*, oral	Ab, CTL CD4+ (Th1), C8+ mediated	Increased levels of IgG2a and 2b P	Zuo SG et al., 2010 [67]

**VEGF**	Xenogeneic DNA vaccine	Fibrosarcoma, breast cancer, hepatoma	"Naked" DNA, IM	Ab CD4+-mediated	Xenopus VEGF has about 75% homology with humans and mice P, T	Wei YQ et al., 2001 [58]
	
	Autologous or xenogeneic protein	Murine and human colon caricinoma; human rhadosarcoma	IM	Ab;	h- or mVEGF conjugated to KLH P	Rad FH et al., 2007 [73]

**FGFR-1/bFGF**	Autologous bFGF peptide	Murine melanoma and lung carcinoma	Lipid A containing liposomes IM	Ab	Effective vaccine against the 44 aa segment of the heparin binding domain; No effect on wound healing or pregancy P	Plum SM et al., 2000 [76] Plum SM et al., 2004 [77]
	
	Xenogeneic DNA vaccine	Murine fibrosarcoma, hepatoma and breast cancer	"Naked" DNA, IM	Ab	FGFR-1 from Xenopus laevis Delayed wound healing P, T	He QM et al., 2003 [78]

**TEM8**	Autologous TEM8 with rat Her2 or human tyrosinase-related protein1 DNA vaccine	Rat Her-2 expressing breast carcinoma; Murine melanoma	Gold-particle gene gun	No Ab or CTL response with TEM8 vaccine alone	Synergy observed P	Felicetti P et al., 2007 [82]
	
	Xenogeneic DNA vaccine	Murine melanoma	*S. typhimurium* oral	CTL;	Human TEM8 No effect on wound healing T	Ruan Z et al., 2009 [83]

**ENDOGLIN (CD105)**	Xenogeneic protein	Murine lung, melanoma, colon carcinoma, fibrosarcoma	SC	Ab;	Synergy with cis-platinum; adjuvant (alum) P, T	Tan GH et al., 2004 [87] Tan GH et al., 2004 [88]

**ANGIOMOTIN**	Xenogeneic DNA vaccine, full-length	Her-2 expressing breast cancer in transgenic mice	Electroporation, TC	Ab	Human angio-motin and Her-2; antitumor synergy when combined with Her-2 DNA vaccine	Holmgren L et al., 2006 [38]

**TIE2**	Xenogeneic protein vaccine	Murine hepatomas and melanomas	SC	Ab	Chicken Tie2 P, T	Luo Y et al., 2006 [94]
	
	DNA vaccine encoding HLA-restricted peptides	*In vitro *lysis of endothelial cells expressing Tie-2; Tumor response not tested	Gold-particle gene gun	CTL	HLA-A*0201/Kb transgenic mice; the epitope (FLPATLTMV) had the highest CTL response;	Ramage JM et al., 2004 [95]

**HP59/SP55**	Xenogeneic peptides	Murine lung carcinoma	Not stated	Ab	HP59 and SP55 peptide mixture P	Fu C et al., 2001 [96]

**Pericyte Targets**						

**HMW-MAA**	Xenogeneic DNA vaccine, HMW-MAA fragment	Murine melanoma, renal carcinoma, Her-2 transgenic mice	*L monocytogenes* IP	Ab, CTL	HMW-MAA (2160-2225 aa) fragment fused to LLO T	Maciag PC et al., 2008 [33]

**PDGFRβ**	Autologous DNA vaccine, full-length	Murine colon, breast, lung carcinoma	*S. typhimurium* oral	CTL	Also, targets activated fibroblasts P, T	Kaplan CD et al., 2006 [34]

**Combined Targets**						

**SURVIVIN**	Xenogeneic DNA vaccine	Murine melanoma	Electroporation, ID	CTL	Human survivin vaccine P	Lladser A et al., 2010 [37]
	
	Survivin/CCL21 DNA vaccine	Murine lung carcinoma	*S. typhimurium*, oral	CTL	Mouse survivin; no effect on wound closure or fertility P, T	Xiang R et al., 2005 [35]

**GRP**	Recombinant chimeric HSP-65 -GRP6 fusion protein	Murine breast carcinomas	SC	Ab, CTL	6 tandem repeats of GRP(18-27 aa) fused to HSP-65 P, T	Guojun W et al., 2008 [115]
	
	Chimeric-HSP65-GRP6 DNA Vaccine	Murine melanoma	"Naked" DNA, IM	Ab	chimera also includes tetanus toxoid and HSP70 fragments; P	Fang J et al., 2009 [116]

**LEGUMAIN**	Allogeneic DNA vaccine	Murine non-small lung, colon and breast cancers	*S. typhimurium*, oral	CTL	Mutant polyubiquitin incorporated P, T	Luo Y et al., 2006 [119]
	
	Autologous DNA minigene	Murine breast carcinoma	*S. typhimurium*, oral	CTL	Angiogenesis inhibited more 90%; H-2K vaccine more potent than H-2D P	Lewen S et al., 2008 [120]

**MMP-2**	Xenogeneic full-length MMP-2 DNA vaccine	Murine fibrosarcoma, hepatoma, lung carcinoma	"Naked" DNA, IM	Ab	Chicken MMP-2 P, T	Su JM et al., 2003 [123]

**β3 Integrin**	Xenogeneic β3 DNA vaccine	Murine fibrosarcoma, mammary carcinoma	"Naked" DNA, IM	Ab	Chicken β3 ligand binding domain P, T	Lou YY et al., 2002 [129]

Abbreviations in table; Ab, antibody response; AP-alkaline phosphatase; CTL, cytotoxic T-lymphocyte response; m, mouse; h, Human; LLO, listerolysin; P, protective vaccine approach in which pre-immunized mice show anti-tumor activity; T, therapeutic vaccine approach in which vaccine, administered after tumor inoculation, has anti-tumor efficacy; keyhole limpet hemocyanin; SC, subcutaneous, ID, intradermal, IM, intramuscular, TC, transcutaneous

Besides tumor cells [41], dendritic cells (DC) pulsed with peptide/protein epitopes (or DNA encoding these epitopes) have also been employed successfully to vaccinate animals against tumor endothelial antigens [42,43]. DC process and present antigens to T and B cells and produce cytokines and chemokines which in turn activate NK cells [44]. DC-based therapies involve modification with pulsed (loaded) defined peptides, whole protein lysate, and/or transfected DNA or RNA [42,43]. An interesting anti-cancer and anti-angiogenic approach was the use of a VEGFR2-loaded DCs that led to greater than 80% reduction in lung metastases of two different tumor models [45]. Different forms of nucleic acids have also been used for angiogenic peptides and proteins. In addition to peptides, proteins, and recombinant DNA, mRNA is another promising strategy to enhance cellular immunity [46]. For example, antitumor synergy was observed when dendritic cells were transfected with mRNA from two receptors (VEGFR-2 and Tie2) that are highly expressed on tumor endothelial cells [47]. Varying the routes of pulsed DC administration may also affect the efficacy of tumor vaccines. Pellegatta and colleagues determined that glioblastomas regressed significantly more when mice received both intratumoral and subcutaneous pulsed DC injections compared to those which received subcutaneous injections [48].

Although most authors have not compared different carrier systems with one another, it is evident that the carrier system and route of administration are critical for the success of the vaccine in animal models and in human clinical trials [49]. We have already discussed differences in the immune response to survivin based on whether electroporation or direct injection of DNA was used. In addition, when Lai et al. compared three different delivery approaches (gene gun with non-coated particles, gene gun with coated gold particles, and intramuscular injection) for the EGFR plasmid vaccine, the gene-gun with non-coated particle vaccine had the greatest cytotoxic T-lymphocyte (CTL) response and anti-tumor response [49]. Interestingly, the CD4+ response and the levels of EGFR-specific antibodies were much greater with the coated gold particle method. The required robust immune response to overcome self-tolerance will no doubt eliminate several carriers, and perhaps autoimmunity will eliminate other carriers. Which of the carriers can be translated successfully from the animal models to humans remains to be determined. The delivery vehicle and the immune-adjuvant will likely be as important as the selected angiogenic antigen to obtain a successful tumor response in humans.

## Approaches for Anti-Angiogenic Vaccines

**Figure 1:**
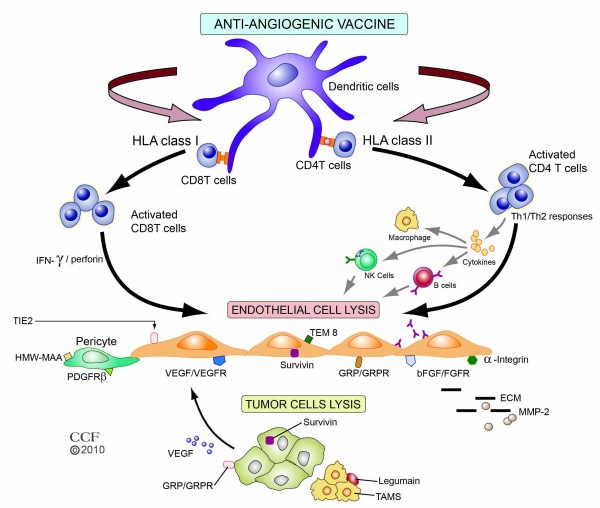
**Major mechanistic immune pathways of anti-angiogenic vaccines and their targets**. Vaccine antigens are processed by antigen processing cells such as dendritic cells and presented to T cells. Depending on the antigen, the route of administration, and the vector, peptide presentation to either major histocompatibility complex (MHC) class I or II occurs, with subsequent interaction with T-cell receptors on CD4+ or CD8+ cells. Cytotoxic CD8+ T cells recognize and lyse tumor endothelial cells directly by perforin-mediated and Fas-mediated cytotoxic mechanisms. CD4+ T-helper cells, through release of different cytokines, can induce Th1 or Th2 responses that stimulate B-cells to produce antibodies and/or activate NK cells and macrophages to inhibit tumor endothelium. Representative targets related to endothelial and cancer cells and their environment for anti-angiogenic vaccines are depicted. Reprinted with permission, Cleveland Clinic Center for Medical Art & Photography © 2010. All Rights Reserved.

A goal of vaccination in anti-angiogenic therapies targeting tumors is to break immune tolerance to self-antigens and induce specific, strong, and persisting immune response leading to eradication of cancer. Complex networks created by several immune-competent cells such as dendritic cells, B cells, cytotoxic CD8+ T, CD4+ T-helper, and NK cells in combination with cytokines, chemokines and other immune mediators are required for effective vaccines and immune reactions against cancer (Figure 1). Two anti-angiogenic vaccine approaches have shown promising results in reducing tumor growth and/or metastases: endothelial cell vaccines that demonstrate antitumor activity and vaccines targeting specific angiogenic targets.

### I. Endothelial Cell Vaccines

#### Whole Cell

In 2000, Wei et al. published a seminal report that demonstrated the efficacy of an endothelial cell vaccine targeting tumors [50]. Studies on tumor models with fibrosarcoma, hepatoma, and breast cancer cells utilized xenogeneic (human or bovine) paraformaldehyde-fixed endothelial cells. In both prophylactic (immunizations followed by tumor inoculation) and therapeutic (tumor inoculation followed by immunization) settings, the tumor growth was markedly inhibited in the three different tumor xenograft models in mice receiving the xenogeneic human umbilical vein endothelial cell (HUVEC) vaccine. In contrast, the syngeneic endothelial vaccine had no effect on the growth of these tumors compared to the phosphate-buffered saline treated control group. Moreover, there was at least a 70% survival of mice injected with the xenogeneic vaccines in the therapeutic setting for at least 80 days post tumor inoculation. Several experiments indicated that the anti-angiogenic and anti-tumor activity in mice immunized with xenogeneic endothelial cells was achieved by induction of CD4+ T lymphocyte-dependent endothelial cell-specific antibodies. Depletion of CD4+ cells abrogated the antitumor activity of the xenogeneic vaccines, whereas depletion of CD8+ and NK cells did not. Moreover, adoptive transfer of immunoglobulins from mice vaccinated with xenogeneic cells resulted in marked tumor inhibition. Further support for the effectiveness of this humoral anti-cancer protection came from *in vitro *studies where antibodies were able to block endothelial cell proliferation and also from *in vivo *experiments where angiogenesis was inhibited in a corneal micropocket assay. These endothelial specific antibodies most likely bind to the vascular endothelial growth factor receptor 2 (VEGFR 2) and α_v_β_3 _integrin.

In addition to the efficacy of xenogeneic vaccines, several studies have demonstrated the anti-tumor efficacy of syngeneic whole endothelial cell vaccines. If an adequate immune response occurs, syngeneic endothelial cell vaccines may be preferable to xenogeneic vaccines because there are fewer species-specific immune reactions. Okaji and colleagues showed that the syngeneic endothelial cell-based vaccine (hepatic sinusosoidal endothelium isolated from BALB/c mice) arrested pulmonary metastases in a murine colon cancer in both protective and therapeutic pre-clinical settings [51]. In addition to induction of inhibitory antibodies that cross-reacted with human and mouse endothelial membrane antigens, this vaccine induced cytotoxic T-lymphocytes specific against endothelial but not tumor cells. In contrast to the previous study of Wei et al [50], the syngeneic cell-based vaccine had modestly greater anti-tumor activity than did the xenogeneic cell vaccine. The reason for disparity between the two studies is not clear, but it might be due to differences in the endothelial cells used in vaccine preparation or the route of administration. Notably, the microvasular hepatic sinusoidal endothelial cells in this study may provide a more effective cell-based vaccine [51]. In the therapeutic experimental vaccine setting, this was the only syngeneic cell vaccine study that showed significant inhibition and/or prolonged survival in the tumor-bearing mice [51]. A second study also demonstrated the efficacy of syngeneic tumor endothelial vaccines. In this preclinical study, Scappaticci and Nolan compared syngeneic, allogeneic, and xenogeneic endothelial cell vaccines for their ability to provide protection against melanoma in a mouse model [52]. The syngeneic endothelial cells were transformed by SV40 T antigen and H-ras, while the allogeneic and xenogeneic hemangioendothelioma cells had transformed spontaneously. Three weeks after the last intraperitoneal (ip) injection of the transformed endothelial cells, the mice were injected subcutaneously with murine melanoma (B16F10) cells. All mice had a humoral immune response to the different endothelial cell vaccines, with vaccinated mice displaying a decrease of 45% in VEGF serum levels compared to control. Furthermore, the group vaccinated with syngeneic cells showed complete tumor inhibition in 50% (3/6) of mice beyond 6 months, whereas mice in untreated and other vaccinated treatment groups died within 3 to 4 weeks post-tumor inoculation. In mice vaccinated with syngeneic cells that eventually developed tumors, microvessel density counts were 4-5 fold lower than were those in the control groups. From adoptive transfer experiments and CD8 knockout mice, the results suggested that both humoral and cellular immunity were important in the syngeneic vaccinated group. Interestingly, the vaccinated long-term survivors were significantly protected from a challenge with a different tumor type (EL-4 lymphoma). More recently, prophylactic vaccination with a syngeneic transformed endothelial cell line, Tpit/E, was shown to inhibit subcutaneous melanoma growth and appearance of lung metastasis in a mouse model [53]. Because death ensued rapidly within 3 to 4 weeks after inoculation of the B16F10 melanomas, therapeutic vaccination was not attempted with this vaccine.

An anti-angiogenic endothelial cell vaccine has also been tested for its anti-tumor activity in a pilot clinical trial of patients who had recurrence of their brain tumors or metastatic colorectal cancer. The patients were treated with glutaraldehyde-fixed human umbilical vein endothelial cells (HUVECs) by intradermal injection every week for the first month and then every 2 weeks [54]. Specific antibodies against HUVEC membrane antigens were detected in eight out of total nine patients and specific cellular immune responses against HUVEC were detected in six of seven tested patients. In addition, magnetic resonance imaging showed partial or complete tumor responses for at least 9 months in three patients with malignant brain tumors, while the three patients with colorectal cancer showed no response. Because there was not a direct correlation between degree of immune response and anti-tumor activity, the authors suggest that further studies are needed to identify factors that facilitate or mute immunity. Nevertheless, the results of this trial were promising in view of the lack of reported side effects and the response to treatment of patients with recalcitrant tumors.

#### Endothelial cell membrane

Instead of using the whole endothelial cells as a vaccine, rat tumor endothelial proteins were isolated from luminal surfaces of vascular endothelial cells containing several up-regulated angiogenesis-associated endothelial proteins [55]. In a rat lung metastatic model, endothelial cell surface proteins were isolated *in situ *by biotinylation followed by strepavidin selection. This mixture of mostly undefined proteins was used as a xenogeneic vaccine to treat subcutaneous LLC tumors in a mouse model. Although the growth of primary tumors was not affected in vaccinated mice, the numbers of lung and liver metastases were reduced by at least 75%. T lymphocytes isolated from immunized mice lysed in a dose-dependent manner endothelial but not tumor cells, confirming specific anti-endothelial properties of this vaccine; adoptive transfer of CTL *in vivo *did not have anti-tumor activity, suggesting that local tumor conditions may suppress their efficacy (e.g., intratumoral regulatory T-lymphocytes). In contrast, adoptive transfer of the IgG antibody from vaccinated into naïve tumor-bearing mice specifically targeted tumor endothelium, reduced metastases, and prolonged life-span in a therapeutic model.

### II. Vaccines Expressing Defined Targets

**Figure 2:**
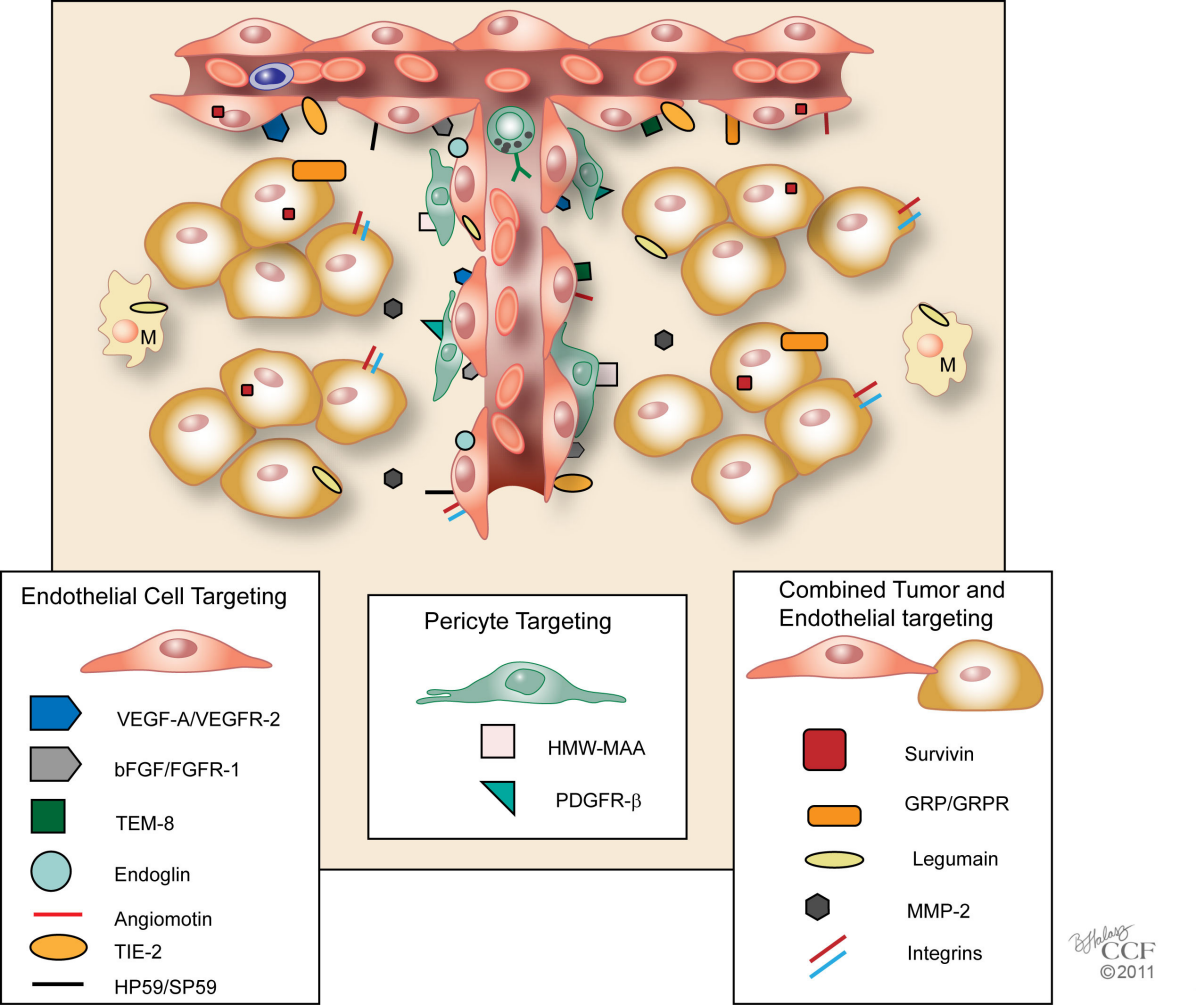
**Tumor Endothelial Vaccines with Defined Targets**. Schematic model of a tumor and its angiogenic vessels are shown with targets of tumor endothelial vaccines. These vaccines may be classified on their specific targets 1) that are primarily associated with tumor endothelial cells, 2) that promote growth of pericytes, or 3) that enhance both tumor and endothelial cell growth or survival. Growth factors were classified based on the location of their receptors. M, Macrophage. Reprinted with permission, Cleveland Clinic Center for Medical Art & Photography © 2011. All Rights Reserved.

Defined endothelial vaccines are based on specific targets and include peptides and nucleic acids (DNA or RNA) that encode these peptides (see Table 1, Figure 2). Suitable angiogenic targets in tumors may be receptors/markers on endothelial cells or alternatively, may be growth factors secreted by cells other than endothelial cells. To date, there has been no target or epitope that is completely specific for tumor endothelial cells. For example, TEM8, one of the more specific tumor endothelial cell markers identified thus far, was originally found in the tumor vasculature and the developing embryo, but it has since been found on cell surfaces of melanomas, breast cancers, and dendritic cells. Despite the overlap in this system, we think that classification of angiogenic vaccines based on preponderance of their targets within most tumors may be useful. As a result, we have divided tumor endothelial vaccines with defined targets into three classes: 1) growth factors/receptors or epitopes that are primarily associated with growth of tumor endothelial cells; 2) growth factors/receptors or epitopes that promote growth of pericytes; and 3) proteins/growth factors/receptors that enhance both tumor and endothelial cell growth or survival. The growth factors were classified, not on their cells of origin, but on the location of their receptors.

#### A. Endothelial cell targets

##### Vascular Endothelial Growth Factor Receptor-2 (VEGFR2)

VEGFR2, also known as FLK-1 or KDR, is an angiogenesis-associated receptor expressed on tumor endothelial cells. Angiogenesis in non-tumor vessels is relatively rare, enabling specific targeting of VEGFR2-mediated tumor angiogenesis with minimal side effects. VEGF-mediated signaling through VEGFR-2 is the key rate-limiting step in tumor angiogenesis, and plays the most important role in neovascularization, development, and progression of various tumors. VEGFR2 also has a central role in physiologic angiogenesis as well as in vasculogenesis as demonstrated by the lack of vasculogenesis in VEGFR2 null mice [56]. VEGFR2 belongs to the tyrosine kinase receptor family that includes VEGFR1, VEGFR2, VEGFR3 and neutropilin 1. Since VEGFR2 is a selective marker for neoplastic endothelium, it has been a target for many approaches, including small molecule kinase inhibitors, synthetic receptor tyrosine kinase inhibitors, monoclonal antibodies, and vaccines [57]. More vaccines and immunization strategies have been developed and tested in pre-clinical models against VEGF/VEGFR2 than with any other tumor angiogenic marker [43,45,58-67], and as a result, VEGF and VEGFR2 targeting will be described separately.

In 2002, Li and colleagues were one of the first groups to establish the utility of a VEGFR-2 vaccine to inhibit tumor angiogenesis and growth [45]. After mice were immunized with DC cells pulsed with a fusion AP-mouse VEGFR2 protein, they were challenged with two different tumors (B16F10 melanoma or Lewis lung carcinoma) to establish pulmonary metastases. In both tumor models, lung metastases were reduced by more than 80% in mice that received this vaccine. Moreover, angiogenesis in the alginate assay was reduced by approximately 65%. Based on depletion of CD4+ and CD8+ T-cell experiments, the anti-angiogenic and anti-tumor action of the vaccine was primarily the result of CD8+ T-cells. Except for reductions in the number of pregnancies and litter size, there were no side effects from the vaccine.

Soon after, Niethammer et al. developed an oral autologous VEGFR2 DNA (pcDNA-3.1-Flk-1) vaccine carried by attenuated *S. typhimurium *targeting tumor angiogenesis [59]. The vaccine significantly inhibited neovascularization in murine melanomas, colon carcinomas, and non-small cell lung carcinomas and as a consequence the vaccine suppressed metastases. In a therapeutic setting in which a colon carcinoma cell line was injected iv, the mouse (m) VEGFR2 vaccine reduced lung metastases by approximately 70%. The endothelial cells expressing VEGFR2 were specifically lysed by CD8+ T-cells from VEGFR2 immunized mice. Whereas pregnancy was not affected, wound healing was modestly delayed with this therapy. More recently, Seavey and colleagues developed an oral listeriolysin-mVEGFR2 DNA vaccine that resulted in marked anti-tumor CTL-mediated inhibition of Her-2-expressing breast cancer tumors (NT-2) in mice [32]. After three listeriolysin-VEGFR2 plasmids were made with non-overlapping mVEGFR-2 fragments, these constructs were incorporated into *Listeria monocytogenes*. Four days after the mice were injected s.c. with NT-2 cells, they received the initial vaccination and by day 64 post tumor inoculation, there was approximately a 70% reduction in tumor size in treatment groups vaccinated with two of the VEGFR2 constructs. There were some mice in the vaccinated group that had no evidence of tumors, but those vaccinated mice that did have tumors had a marked reduction in the microvessel density of these tumors. Most interestingly, the anti-tumor response from the mVEGFR2 vaccine was not solely dependent on vascular targeting. The vaccine induced a CTL-mediated response against the Her-2 antigen on tumor cells which the authors attributed not to cross-reactivity but to epitope spreading of Her-2. Epitope spreading was hypothesized to be the result of destruction of tumor cells and release of tumor antigens (e.g., Her-2) owing to reduction in angiogenesis. There was no effect on wound healing, and, in contrast to an earlier study [45], the vaccine had no toxic side effects on pregnancy.

Compared to large full-length coding VEGFR2 DNA insert (> 4000 base pairs), a minigene DNA vaccine with DNA encoding MHC-I-restricted peptides is a particularly attractive approach to minimize side effects. Utilizing a restricted minigene approach in which five H2-D^d ^and/or H-2K^d ^restricted peptides were inserted per plasmid construct, Luo et al. determined that an oral vaccine delivered by *S. typhimurium *protected against tumors of different origin (D2F2, CT-26) in BALB/c mice [63]. In a prophylactic experimental design, the survival rate in vaccinated mice was about 4-fold greater than in the control group. Some of the minigenes tested had anti-tumor activity equivalent to the full-length VEGFR2 vaccine. The vaccine was specific to VEGFR2 proliferating endothelial cells and lysed them in CTL-specific manner. In a similar study, Zhou et al. demonstrated the anti-tumor efficacy of VEGFR2 minigene vaccine targeting H-2D^d^-restricted VEGFR2 epitopes in a different mouse strain (C57BL/6J) [61].

Many of the advantages that the minigene approach offers are shared by the peptide vaccine approach. That is, the small sizes of the peptides are likely to have less toxicity and the sequences of the peptides can easily be modified to optimize binding to MHC-I-like molecules. Using binding algorithms and MHC binding assays, two naturally processed CD8 T-cell epitopes (VILTNPISM and FSNSTNDILI) were identified from murine VEGFR2 [62]. Cytotoxic T lymphocytes targeting endothelial cells effectively reduced angiogenesis in a Matrigel assay and inhibited tumor growth by approximately 50% in a therapeutic setting in mice [62]. Another study identified a potent immunogenic epitope, VEGFR2-169 peptide (RFVPDGNRI), that was able to evoke strong CTL responses in cancer patients [43]. Indeed, the epitope VEGFR2-169 vaccine in combination with gemcitabine was tested in a phase I clinical trial for patients with metastatic and unresectable pancreatic cancer [66]; 83% of patients who completed one course of treatment (4 vaccinations) developed immunological reactions at the injection sites and in 61% patients, specific CTLs against the vaccination peptide were detected. No severe side reactions of grade 4 or higher were noted in the clinical trial and grade 3 toxicities were caused by non-vaccine therapies (e.g., gemcitabine). On the basis of not identifying a dose-limiting toxicity in this trial for the VEGFR-169 vaccine, the optimal dose in future clinical trials should be 2 mg per individual or higher.

The results of two other studies will be discussed in which cationic liposomes were the carriers and adjuvants. Xie et al determined that a xenogeneic human VEGFR2 DNA vaccine had marked anti-tumor and angiogenic activity *in vivo *[65]. In the therapeutic setting in which mice had established murine breast or colon cancers, 60% of the group receiving the xenogeneic vaccine survived for 2 months, while all mice in the control (dextrose-treated) group died by day 40. The VEGFR2-containing plasmid was incorporated within cationic liposomes and injected iv once a week for six injections. In contrast to previous studies, the murine VEGFR2 had little protective or therapeutic activity on tumor size or growth in this study. This may be due to use of different DNA delivery vectors, chemical/genetic adjuvants and/or routes of administration. In a second study, cationic liposomes were also used to incorporate a plasmid with a fusion of murine β-defensin 2 (MBD2) and murine VEGFR2 to immunize tumor-bearing mice [64]. MBD2 is an antimicrobial peptide, which augments innate and adaptive immunity by activating Toll-like receptor 4 and recruiting immature dendritic cells [68,69]. Three days after Lewis Lung tumor cells were injected iv, the mice received the first of four intramuscular injections of the vaccine. Compared to the murine VEGFR2 vaccine, the fusion vaccine resulted in mice with 50% fewer lung metastases and in tumors with reduced angiogenesis. Clearly, the fusion protein attached to the VEGFR2 greatly enhanced the activity of this vaccine.

##### Vascular Endothelial Growth Factor-A

Based on alternative splicing, there are five pro-angiogenic variants of vascular endothelial growth factor (VEGF-A) that target and activate VEGFR1 and VEGFR2. The function of each of these different forms of VEGF in tumor angiogenesis is not completely known, but larger molecular weight isoforms of VEGF-A (VEGF-A165, VEGF-A189) in solid tumors may be associated with increased angiogenesis, metastases, and a worse prognosis [70,71]. In addition, VEGF is secreted by both tumor and endothelial cells, but despite its origin, the primary target of VEGF-A is its cognate receptors located on angiogenic vessels.

Several different VEGF immunization vectors, including plasmids, T7 recombinant phage, and proteins, have demonstrated anti-tumor efficacy based on an antibody-dependent response [58,72,73]. A DNA vaccine with the *Xenopus *vascular endothelial growth factor was used to induce anti-angiogenic and anti-tumor activity in a mouse model [58]. *Xenopus *VEGF has an approximately 75% amino acid homology with VEGF from mice and humans, making it a good vaccination candidate to break tolerance. In both prophylactic and therapeutic settings, this vaccine administered intramuscularly was effective against several tumor-bearing models (MA 782/55 breast cancer, Meth A fibrosarcoma, and H22 hepatoma). In mice that were injected sc with tumor cells 7 days before immunization, the survival rate at 90 days was between 50 and 60%, while mice from control groups died before 50 days. Indicative of the central role of CD4+ T-cells with this vaccine, adoptive transfer of sera containing VEGF antibodies blocked tumor growth and angiogenesis *in vivo*. Moreover, the anti-tumor activity from the *Xenopus *VEGF vaccine was completely eliminated with depletion of CD4+ cells, while depletion of CD8+ and NK cells had no effect.

Similar to the previously discussed VEGF DNA vaccine study [58,72,73], the "VEGF kinoid" vaccine, composed of murine (m) or human (h) VEGF and keyhole limpet hemocyanin, triggered a potent anti-VEGF antibody response [73]. The (h, m) "VEGF kinoid" vaccine was prepared by complexing VEGF with the immunogenic carrier, limpet hemocyanin, in the presence of glutaraldehyde. In BALB/c mice immunized with VEGF-kinoid, the number and size of lung metastases challenged with a colorectal tumor were significantly decreased. *In vitro*, the anti-VEGF antibodies from immunized mice inhibited both proliferation of cultured HUVECs and the binding of mVEGF to its receptor, VEGFR2. Moreover, BALB/c mice immunized with hVEGF kinoid had high levels of hVEGF antibodies in serum, and adoptive transfer of purified IgG from these mice decreased by more than 50% the size of tumors from different origins (human A673 rhabdomyosarcoma, HT29 colon carcinoma) in immunocompromised mice.

Notably, there are anti-angiogenic isoforms of VEGF-A that differ from their angiogenic counterparts at the C-terminal end [74,75]. Although the angiogenic isoforms likely dominate in more angiogenic tumors, there is probably a balance between these isoforms in less vascular tumors [75]. Consideration of these anti-angiogenic isoforms of VEGF-A in vaccine development has thus far been lacking. With further development of vaccines against VEGF-A, avoidance of targeting these anti-angiogenic forms will likely have an important role in obtaining greater efficacy and specificity of the vaccine with fewer undesired toxic effects. In addition to targeting VEGF-A, other VEGFs such as VEGF-C and -D that promote tumor angiogenesis and lymphangiogenesis hold promise but as of yet are untested vaccine targets.

##### FGFR-1/bFGF

Fibroblast growth factor receptor-1 (FGFR-1, CD331) is a high affinity receptor for basic fibroblast growth factor (bFGF, FGF2) and the signal transduction pathway induced by the bFGF/FGFR-1 interaction has an important role in stimulating tumor angiogenesis. Both bFGF and FGFR-1 have been targeted by vaccines to reduce tumor size in pre-clinical mouse models. Plum et al compared liposomal autologous bFGF peptide vaccines targeting either the heparin binding domain (HBD) or the receptor binding domain (RBD) of the growth factor [76]. Although the RBD vaccine had no anti-angiogenic or anti-tumor effect, the HBD vaccine showed marked anti-angiogenic activity in a gelfoam sponge model as well as anti-tumor activity. In a metastasis model of murine B16 melanoma or Lewis lung carcinoma cells, the HBD vaccine decreased pulmonary metastases by more than 90%. Furthermore, the primary anti-angiogenic mechanism of this vaccine was the production of anti-FGF antibodies [76]. Notably, this vaccine did not interfere with wound healing or with reproduction [77].

Alternatively, He and colleagues used a xenogeneic plasmid DNA vaccine to target FGFR-1 and to break the immune tolerance [78]. The amino acid sequence of *Xenopus *FGFR-1 has homology of 74% and 80% with human and mouse receptor, respectively. Intramuscular vaccination of the *Xenopus *FGFR-1 plasmid before injecting tumor cells subcutaneously (Meth A fibrosarcoma, H22 hepatoma, or MA782/5 S breast cancer) led to an 80% or greater reduction in tumor size compared to the control [78]. When the FGFR-1 vaccine was administered 7 days after inoculation of tumor cells, the mice had prolonged survival with significantly reduced tumor size compared to controls. Whereas all the mice injected with the three different tumors in the control groups had died by day 50 after tumor cell injection, 80% of the mice in the FGFR-1-vaccinated group were still alive. The vaccine induced antibodies specific toward tumor endothelial cells and the *in vivo *anti-tumor effect could be eliminated by depletion of CD4+ T-cells. Except for delayed wound healing, there was no observed toxicity as measured by weight loss, behavior, life-span or histology.

##### TEM8

With the serial analysis of gene expression approach, tumor endothelial marker 8 (TEM8) was identified as a specific cell surface marker for tumor vessels. The physiologic function of TEM8 is not known, although there is a specific interaction between TEM8 and the α3 subunit of collagen as determined by the two-hybrid assay [79]. In contrast to most other endothelial markers and/or receptors identified thus far, the mRNA of TEM 8 was undetectable in the endothelium of healing wounds or in the corpus luteum [12]. Consequently, TEM8 is a very specific marker for tumor angiogenesis and is an ideal candidate for vaccine development. Nevertheless, TEM8 is expressed in endothelial cells of the developing embryo [80] and lung endothelium [13], dendritic cells [81], melanomas, and breast cancer [82].

Felicetti and colleages were the first group to develop a TEM8 DNA vaccine [82]. Although the syngeneic TEM8 vaccine injected into the stroma of rat Her2-expressing breast cancer (233-VSGA1) was ineffective when used alone, the combination of TEM8 and rat Her-2 DNA vaccine was synergistic in reducing tumor growth and extending tumor-free survival. Similarly, the combination of TEM8 and tyrosinase-related protein-1 DNA vaccines were synergistic in their anti-tumor activity against B16F10 melanomas.

In contrast to previous studies, Ruan and colleagues developed a TEM8 vaccine that did have anti-tumor activity. In this study, a xenogeneic DNA vaccine encoding human TEM8 carried by attenuated *S. typhimurium *was tested for its anti-tumor activity in mice with B16F10 melanoma xenografts [83]. Oral administration of the vaccine post-tumor challenge significantly decreased tumor volume and prolonged life-span of vaccinated mice. Whereas all mice had died at day 30 in the control group injected iv with melanoma cells, none of the mice in the TEM8-vaccinated mice had died and 60% of the mice were alive at day 60. Additionally, experimental pulmonary metastases were markedly reduced compared to those in the control group. Experimental evidence showed that the CD8 T-cell population was mainly responsible for the immune response. Notably, no toxicity from the TEM8 vaccine was observed as evidenced by normal wound healing and neurological testing (fertility was not examined in this study). Because of the finding that the expression of TEM8 is increased in endothelial cells in the lungs of mice [13], more detail studies are required to determine the safety of this vaccine.

##### Endoglin (CD105)

Endoglin, a 95-kDa cell surface protein expressed as a homodimer, functions as an accessory protein for kinase receptor complexes of the TGF-β superfamily and modulates TGF-β signaling. Expression of endoglin is correlated with vascular density and poor prognosis in patients with breast and colorectal carcinomas [84,85]. In addition to its expression on selected tumors, endoglin is over-expressed on proliferating endothelial cells in tumors and thus offers an attractive target for anti-angiogenic therapy [86]. Tan et al. developed a xenogeneic (porcine) endoglin protein vaccine administered in a tumor-bearing mouse model that induced protective and therapeutic anti-tumor immunity. The vaccine inhibited tumor growth and prolonged survival in a range of tumors including Lewis lung carcinoma, B16 melanoma, CT26 colon carcinoma, and Meth A fibrosarcoma. Deletion of CD4(+) T-lymphocytes abrogated the anti-tumor activity and endoglin-specific autoantibodies [87]. Moreover, the combination of low-dose cisplatin with the endoglin vaccine showed at least additive anti-cancer and anti-angiogenic effects [88]. While all murine CT26 carcinoma-bearing mice survived to day 50 in the combined vaccine-chemotherapy group, 60% and 70% of the chemotherapy and vaccine-alone treated mice, respectively, were alive. No toxicity was observed in the mice treated with the vaccine or the vaccine-chemotherapy groups. For instance, there was no difference in weight, life-span, liver enzymes, white or red blood cells counts, or tissue histology between the combined vaccine-chemotherapy and the untreated groups.

##### Angiomotin

The shorter splice variant of angiomotin, p80 that is a membrane-associated protein is expressed in tumor and placental endothelium and mediates endothelial cell migration *in vitro *[89]. The anti-angiogenic peptide, angiostatin, has high affinity with angiomotin and inhibits its function by reducing endothelial cell migration and tube formation *in vitro *[89,90]. The role of angiomotin in endothelial cell migration and its correlation with poor survival in breast cancer patients [91] suggest that it is a potential target for anti-angiogenic immunotherapy. Indeed, targeting angiomotin by DNA vaccination efficiently inhibited angiogenesis and tumor growth *in vivo*. Since xenogeneic proteins frequently break tolerance against self-antigens, a plasmid expressing the human p80 isoform of angiomotin was administered to different tumor-bearing mouse models. In an experiment in which the BALB/c mice were electroporated transcutaneously with the angiomotin construct before their challenge with breast cancer cells, there was complete suppression of tumor growth in 12 of 18 mice more than 150 days [38]. In a similarly designed experiment but in knockout B-cell mice, the anti-tumor efficacy of the angiomotin vaccine was lost, indicating that CD4+ cells and a humoral immune response were essential. In a Her2/neu mouse model in which spontaneous breast cancers develop, there was no difference in the number of tumor-free mice between the angiomotin-vaccinated and the control mice; all mice in both groups had tumors between the 20 and 25^th ^week of age. However, mice treated with a combination of angiomotin and Her-2 vaccine were 80% tumor-free compared to 20% of mice treated with the Her 2 vaccine alone when followed up to 70 weeks after birth. In a Matrigel angiogenic assay, the number of blood vessels was significantly reduced in mice treated with the angiomotin vaccine. No toxicity was observed with the angiomotin vaccine in mice more than 1 year after it was administered.

##### TIE-2

Tie-2 is a receptor tyrosine kinase that is over-expressed in activated tumor endothelial cells and has a role in stabilizing interactions between pericytes and endothelial cells in the tumor vasculature [92]. Moreover, administration of adenovirus expressing soluble Tie-2, which is able to block activation of Tie-2, inhibited growth of primary tumors and metastases [93]. These results suggest that Tie-2 could be a target for a therapeutic vaccine and two studies suggest the potential of such a vaccine.

To overcome tolerance, a protein vaccine based on the chicken Tie-2, which has about 70% amino acid homology with mice and humans, was tested for its anti-tumor and anti-angiogenic activity in a tumor-bearing mouse model [94]. Both prophylactic and therapeutic vaccine approaches with chicken-based TIE-2 reduced growth of subcutaneous H22 hepatomas and B16F10 melanomas, while vaccination with the mouse TIE-2 had no effect on growth. Furthermore, mice that were immunized with the chicken TIE-2 vaccine 7 days after tail vein injection of B16 melanomas had a 75% reduction in metastases. Indicative of a CD4+-mediated response, adoptive transfer of purified antibodies from the immunized mice results in an anti-tumor response *in vivo *and apoptosis of endothelial cells *in vitro*. Moreover, depletion of CD8+ and NK cells did not alter the efficacy of the vaccine whereas depletion of CD4+ cells eliminated its anti-tumor activity. In addition to the apoptotic effect of antibodies on endothelial cells *in vitro*, microvessel density in the tumor and in the alginate encapsulation assay was reduced indicating that anti-angiogenesis was the primary effect of the vaccine.

In contrast to this study, Ramage et al. used a CD8+-mediated strategy to induce HLA-A*0201-restricted endothelial-specific CTLs in mice immunized with autologous Tie-2 DNA vaccine [95]. Because HLA-A*0201 was one of the most frequent HLA-A alleles in humans, Tie2 was screened for HLA-A*0201 binding epitopes; four such peptides were identified and modified by amino acid substitution (Z84, Z95, Z101, and Z107) to enhance HLA binding and immunogenicity of these epitopes. After plasmid constructs expressing the Tie-2 epitopes were made, HLA-A*0201 transgenic mice were immunized with these constructs administered by a gene gun. The construct containing the Z84 epitope was most efficient in generating CTLs that were able to kill human endothelial cells over-expressing Tie-2. The anti-tumor activity of this vaccine approach has not been reported.

##### HP59/SP55

SP55 and its human homolog, HP59, are transmembrane proteins that share 86% homology [96]. HP59 is present in lung endothelium until 5 days after birth [97,98], but after the neonatal period, HP59 is not detected in normal vasculature of humans [96]. Nevertheless, HP59 is up-regulated in the vasculature of solid tumors and rheumatoid arthritis. CM101, a polysaccharide isolated from Group B streptococcus, is a ligand for these two proteins and inhibits a number of tumors [96]. It is unlikely that HP59 or SP55 proteins are increased in wound healing since their ligand, CM101, does not affect wound healing. As a result of these findings of the selective increase of HP59 in tumor vessels, Fu et al examined whether a xenogeneic peptide vaccine from HP59/SP55 would inhibit tumor growth in a mouse model [96]. After receiving the HP59/SP55 peptide vaccine, the mice were challenged with Lewis lung carcinoma cells; compared to controls, the vaccinated mice had a tumor burden that was 38% of control. Immunohistological evaluation of the tumors indicated that the SP55/HP59 vaccine inhibited not only tumor angiogenesis but also vasculogenesis.

#### B. Pericyte targets

##### HMW-MAA

High Molecular Weight Melanoma-Associated Antigen (HMW-MAA) is a surface chondroitin sulfate proteoglycan with restricted distribution in normal tissue, but which is expressed in a wide range of tumor tissues and is involved in progression and development of metastases of melanoma cells. This antigen can be used to target angiogenesis since HMW-MAA is abundantly expressed on activated pericytes, which have an essential role in neovascularization of tumors. Maciag et al. generated a recombinant *L. monocytogenes *(*Lm*-LLO-HMW-MAA-C) that contains and secretes a fragment of HMW-MAA (residues 2,160-2,258) fused to the first 441 residues of the listeriolysin O (LLO) protein [33]. Three days after mice were injected with B16F10 melanoma cells that expressed full-length HMW-MAA, the mice were immunized intraperitoneally with Lm-LL0 HMW-MAA-C vaccine; 62.5% (5 of 8) of mice were tumor free on day 56 after tumor inoculation while no mice in the control groups remained tumor-free. The immunized mice that were tumor-free developed long-term immunity as demonstrated by the rejection of a second lethal dose of the melanoma cell line. Whereas the control mice had developed tumors 10 days after tumor inoculation, more than 90% of *Lm*-LLO-HMW-MAA-C vaccinated mice rejected the cell line. Depletion of either CD8+ or CD4+ cells abrogated the protective effect of the vaccine, indicating that both cell types were participating in the anti-tumor immunity. Notably, *Lm*-LLO-HMW-MAA-C vaccine displayed anti-tumor effects against several other cancers (the parent line, B16F10; kidney cancer, RENCA; a teratocarcinoma NT-2) that did not express HMW-MAA. In mice with HMW-MAA negative tumors treated by the vaccine, pericytes were significantly depleted in these tumors. These findings suggest that pericytes in many tumors are the likely target of the vaccine.

##### PDGFR-β

Another potential target over-expressed on tumor pericytes is platelet derived growth factor receptor beta (PDGFR-β). This signaling pathway is activated in physiological processes such as embryonic development and wound healing but this pathway is also up-regulated in cancers [99,100]. In both tumors and embryos, angiogenic endothelial cells expressing PDGFβ recruit pericytes to stabilize and remodel vessels. Furthermore, Song and colleagues determined that reduction of PDGFRβ-expressing pericytes led to endothelial cell apoptosis, vascular dilation, and decreased tumor volume in pancreatic islet tumors in mice [101].

On the basis of these results, a strategy targeting pericytes with a PDGFRβ-based DNA vaccine was developed by Kaplan et al. [34]. This oral vaccine, delivered by transformed *S. typhimurium*, reduced the number of tumor-associated pericytes and protected mice from colon, breast and lung carcinomas. In both prophylactic and therapeutic settings, the tumor burden was reduced by at least 60%. Moreover, in tumors implanted in mice 20 days before the oral vaccine was administered, the vaccine was still surprisingly effective: the size of tumors in the PDGFRβ vaccine-treated group was about one-fourth the size of the control groups (198 mm^3 ^vs. 898 mm^3^). Although the intratumoral VEGF levels were unchanged with the PDGFRβ vaccine, the angiogenic marker, isolectin B4-FITC, was significantly decreased in bFGF-supplemented Matrigel assay. Based on standard Cr-release assays from this study, the mechanism of the tumor inhibition by the vaccine was due to a PDGFR-β-specific CTL immune response.

#### C. Vaccines that target both endothelial and tumor cells

##### Survivin

Survivin is an intracellular protein that inhibits apoptosis. Although survivin is normally expressed during embryonic development, it is not expressed in differentiated cells. Nevertheless, survivin is up-regulated in human cancer and tumor-associated endothelial cells to avoid apoptosis. Thus, therapies including vaccines targeting survivin induce apoptosis of tumor cells and their angiogenic vessels [35,37,39,102]. In an early study, naked DNA vaccines against full-length survivin administered intramuscularly were found to have effective humoral and cellular immune responses in mouse models [39]. Although the anti-tumor efficacy was not tested in this study, Lladser's group later reported that naked DNA vaccination with survivin was less effective in preventing tumors from developing in either the prophylactic or therapeutic settings [37]. More effective delivery systems for survivin vaccines have since been developed. In a later study, Lladser's group delivered the human survivin DNA vaccine by intradermal electroporation [37]. Against mouse B16 melanoma tumors, a xenogeneic DNA vaccine encoding human survivin induced a cross-reactive cytotoxic response toward the mouse survivin 20-28 epitope, suggesting that this vaccination was able to break self-tolerance. In an *in vivo *angiogenic Matrigel assay, the vascular density decreased nearly 50% in the survivin-vaccinated mice compared to the plasmid control-vaccinated mice. In mice immunized with survivin before tumor inoculation, 4 of 10 mice remained tumor-free for 100 days while all vaccinated control mice developed tumors and had died by day 30 after tumor cell challenge. In a second experimental design in which the mice received their first immunization 10 days after sc injection of B16 cells, there was also a 40% protection in the survivin-vaccinated group. In contrast to intradermal electroporation, naked DNA vaccination with full-length survivin showed minimal protection against tumor development.

An oral vaccine delivery system for a plasmid that expressed survivin and the CCL21 chemokine was developed by Xiang et al [35]. CCL21 may synergize with the survivin target through a number of its functions. By attracting activated dendritic cells, enhancing T- cells mediated immune responses, and inhibiting angiogenesis, CCL21 through binding to its primary receptor CCR7 and to CXCR3 may synergize with the survivin target. To optimize epitope processing by proteasomes and antigen presentation to MHC-I, a mutant polyubiquitin was included in the plasmid construct. The vaccine was delivered orally by an attenuated double mutated *Salmonella *strain (dam^- ^and AroA^-^) to eight mice with D121 murine Lewis lung carcinoma. In the prophylactic setting, six mice that received the survivin-CCL21 vaccine had no evidence of pulmonary tumor metastases, while two other mice had significant suppression of tumor metastases. In contrast, seven of eight mice that received the survivin alone vaccine developed metastases as did all other groups (PBS, empty vector, and CCL21 alone treatment group). A similar efficacy pattern was observed with the therapeutic experimental design with the greatest inhibition observed in mice that were vaccinated with survivin-CCL21 vaccine. Mechanistic studies revealed that survivin-CCL21-based vaccine triggered CTL-mediated tumor cell apoptosis and significantly suppressed angiogenesis in the tumor mass. In a Matrigel assay that examined the anti-angiogenic effects of different treatments in the absence of tumor cells, the survivin-CCL21 vaccine-treated group reduced blood vessel density by approximately 3-fold more than the control groups. Moreover, CD8+ T-cells isolated from the survivin-CCL21 treated group specifically lysed murine endothelial cells that expressed survivin *in vitro*. Although the survivin-CCL21 vaccine group markedly inhibited tumor angiogenesis, it is of interest that angiogenesis of wound healing and fertility were unaffected.

##### Gastrin Releasing Peptide/Gastrin Releasing Peptide Receptor

Gastrin Releasing Peptide (GRP), a 27-mer amino acid peptide, is an autocrine/paracrine growth factor that belongs to the bombesin peptide family of neuropeptides involved in many steps of tumor progression including angiogenesis. GRP is a strong mitogen that augments the growth of several types of tumors including breast, prostate, pancreatic, small cell lung, carcinoids, and neuroblastomas [103-110]. By activating its receptor (GRPR) located on both tumor and endothelial cells, GRP is also a direct angiogenic factor as shown by *in vitro *and *in vivo *angiogenic assays [111]. In addition, GRP is able to contribute indirectly to tumor angiogenesis by augmenting expression of pro-angiogenic factors such as IL-8 and VEGF [112,113]. The GRP antagonist, 77427, markedly inhibits endothelial cell cord formation *in vitro *and inhibits angiogenesis *in vivo *[111]. Several therapeutic strategies targeting GRP/GRPR, such as peptide antagonists and monoclonal antibodies, have demonstrated their antitumor efficacy *in vitro *and *in vivo *[114].

Recently, vaccines targeting GRP displayed an effective anti-tumor response by inducing humoral and cell-mediated immune responses. In this study, a recombinant chimeric protein vaccine (HG6) containing six tandem repeats of a GRP fragment (18 to 27 amino acids) fused to the 65-kDa heat shock protein (HSP65) was used to treat mice challenged with breast cancer [115]. The mycobacterial HSP65 was used to enhance humoral immunity. In mice that were vaccinated with the GRP-fusion HG6 protein before inoculation of breast cancer cells (EMT-6), there was a 3-fold reduction in tumor size compared to that of the control groups (phosphate buffered saline or HSP65-vaccinated mice). Similarly, the GRP reduced tumor size (approximately 2-fold) significantly more than the control groups when mice received the vaccine after-tumor inoculation. Also, there were fewer blood vessels around each implant site from HG6-immunized mice (37 vessels) than those from the control-treated mice, PBS (125 vessels) and HSP65-immunized mice (113 vessels). In an analogous approach, a DNA vaccine encoding the GRP-HSP65 fusion product showed anti-tumor activities in a prophylactic setting for subcutaneous implanted tumors and a pulmonary metastasis model [116]. Although the receptor of gastrin-releasing peptide is a promising target, there have been no pre-clinical studies on the utility of such a vaccine. Nonetheless, there have been non-vaccine therapeutic agents showing antitumor efficacy that have targeted GRPR in mouse experiments [114].

##### Legumain

This hypoxia-induced stress protein is an asparaginyl endopeptidase that is over-expressed in tumors, particularly in tumor-associated macrophages (TAMs) but also in tumor endothelial cells and tumor cells [117-119]. Legumain is located in the endosomal/lysosomal pathway and cell surfaces of cancer cells, tumor endothelial cells, and TAMs [117]. In contrast to M1 macrophages that do not express legumain and have immune-surveillance anti-tumor activity, most TAMs are polarized M2 macrophages that promote tumor growth and angiogenesis. When pro-tumor growth mediators such as IL-6, IL-10, MMP-9, and VEGF are the primary secreted factors from TAMs, then tumor angiogenesis, enhanced cell motility, and increased tumor cell invasion are facilitated.

Luo et al. [119] constructed a DNA vaccine encoding a legumain-mutant polyubiquitin fusion protein that was incorporated within an attenuated *S. typhimurium*. The vaccine was then tested in prophylactic and therapeutic experimental models. In the prophylactic experiment, 1 week after receiving the last oral immunization, malignant cell lines (breast, colon, and non-small lung) were injected intravenously in the syngeneic mouse model. Twenty-four days later, the number of lung metastases was determined to be significantly lower in the legumain-vaccinated mice. In the therapeutic experimental setting, the mice were first challenged with breast cancer cells (4T1) implanted subcutaneously and then immunized with a legumain-based vaccine. After primary tumor resection 12 days after initial injection, the mice were followed up for 3 months. While 75% (6/8) of immunized mice survived for 3 months, all mice in the control groups were dead by 1 month; moreover, 62% (5/8) of the immunized mice were totally free of metastases. Specific CD8+ CTL response toward TAMs and depletion of CD8+ cells indicate that the efficacy of the vaccine was primarily mediated by CD8+ cells. These results suggest that legumain-based DNA vaccine induced specific cytotoxic CD8+ T-cell responses by initially activating DCs in Peyer's patches, followed by MHC class I peptide presentation. Vessel growth was significantly reduced with the legumain vaccine with marked reduction in proangiogenic factors released by TAMs such as TGF-β, TNF-α, MMP-9, and VEGF. By removing the primary tumor, this vaccine strategy mirrors a suggested vaccine approach for humans with metastatic disease.

An alternative to the full-length legumain gene vaccine is the approach utilizing legumain minigene vaccine [120]. The potential advantage of minigenes over the full-length legumain is their ease of synthesis and the lack of irrelevant antigenic epitopes, which can cause serious side effects. On the basis of the binding predictions of peptides to the MHC class I, two minigenes encoding legumain H-2D or H-2K restricted epitopes were evaluated for their anti-tumor and anti-angiogenic effect. Although both minigenes showed anti-tumor activity, the H-2K minigene vaccine showed more activity. In a prophylactic setting, the H-2K minigene vaccine inhibited local tumor growth (D2F2 breast cancer cells) and metastases by approximately 50% and 75%, respectively, compared to empty vector control. In addition, the H-2K minigene induced a specific and potent anti-tumor immune response by activating CD8+ T-cells. Similar to the full-length legumain vaccine, the minigene strategy abrogated TAMs from the tumor, thereby reducing proangiogenic factors, which have key roles in tumor angiogenesis. Notably, in a Matrigel assay which was not dependent on the presence of tumor cells, the H-2K minigene vaccine greatly reduced angiogenesis by more than 90%. Unlike the full-length vaccine, the minigene vaccine was not tested in a therapeutic setting.

##### MMP-2

MMP-2 (Matrix Metalloproteinase-2) belongs to the MMP-family of enzymes responsible for tissue remodeling, thereby allowing endothelial cells to migrate and form new blood vessels [121]. Several studies have shown a correlation between elevated levels of stromal MMP-2 and increased aggressiveness and metastases of some tumors [122]. Because there is high homology in amino acid sequences of MMP-2 among chicken, mice, and humans, a xenogeneic chicken MMP-2 was used as the basis for a vaccine to overcome tolerance in treating cancers in mice and humans [123]. Su et al. determined that a DNA vaccine encoding chicken MMP-2 had protective and therapeutic anticancer activity against murine fibrosarcoma, hepatoma, and lung carcinoma. In contrast, vaccination with the mouse MMP-2 had no anticancer activity. In the prophylactic setting, there was significant inhibition of tumor growth and prolonged survival in mice who received the cMMP-2. There was also a 50 to 77% reduction in lung metastases in the cMMP-2 vaccine-treated mice. In the therapeutic setting, there was a 40 to 50% survival rate among tumor-bearing mice at day 50 while all mice in control groups (including those that had received the mouse MMP-2 vaccine) had died by day 40. Depletion of CD4+ cells in tumor-bearing mice eliminated the anti-tumor efficacy of the cMMP-2 vaccine, whereas depletion of CD8+ or NK cells had no effect. Further evidence that the anti-tumor effect was mediated by CD4+ cell activation came from adoptive transfer experiments in which purified antibodies from cMMP-2-vaccinated mice blocked transmigration of tumor and endothelial cells *in vitro*, inhibited angiogenesis in the chick chorio-allantoic membrane assay, and reduced tumor growth *in vivo*. Although this pre-clinical study shows promise, earlier clinical trials with MMP inhibitors have been disappointing, perhaps because of their broad spectrum of inhibitory activity [124-126]. In addition to MMP-2, pre-clinical experiments investigating the efficacy of vaccines targeting MMP-1 and -7 for their anti-tumor activity warrant consideration [127].

##### Integrins

Neovascular endothelial cells in tumor tissues express proteins not present or not readily detectable in quiescent vascular endothelial cells, such as α_v_β_3 _integrins. Integrins, comprised of α and β subunits, are heterodimeric cell-surface-adhesion receptors that bind to the extracellular matrix (ECM) and that are essential for tumor cell and endothelial cell growth. The α_v_β_5 _and α_v_β_3 _are key integrins in promoting angiogenesis and several inhibitors directed against these integrins have shown an anti-angiogenic effect [128]. A xenogeneic DNA vaccine encoding the ligand-binding domain of chicken integrin β3 was tested for its anti-tumor activity in several tumor models in mice [129]. In both protective and therapeutic immunity models, the vaccine demonstrated anti-tumor activity through induction of CD4+-dependent antibodies. Purified immunoglobulins against β3 integrin inhibited endothelial cell proliferation *in vitro *and possessed anti-tumor and anti-angiogenic activity by adoptive transfer *in vivo*.

#### Adverse Effects

Except for delayed wound healing and decreased fertility, investigators in the vast majority of studies reported no morbidity in mice administered vaccines targeting endothelial cells [38,47,51,62,73,78,82,94,96]. These studies ranged from endothelial cell vaccines to defined endothelial targets such as MMP-2, VEGFR2, FGFR-1, or endogolin [50,51,87,123]. Vaccinated mice targeting the tumor endothelium were noted to have a normal physical appearance, normal life span, no weight loss, no signs of distress, no bleeding disorders, and have no pathological changes in their tissues [50,55,59,83,87,115,123,130]. In only one study did the vaccinated mice appear to show a change in their physical habitus: in this report, mice vaccinated with DC pulsed with a VEGFR2 peptide had weight loss, ruffling of the fur, and a change in their behavior [65].

Because cross-reactivity with physiologic angiogenic targets was a primary concern with this vaccination strategy, wound healing and fertility were carefully investigated in several studies. Some vaccines targeting VEGFR2, FGFR-1, and endogolin resulted in delayed wound healing [43,59,78,87]. Niethammer et al specifically commented about the delay in wound healing which was quite modest in vaccinated mice -- 13 days for control mice vs 14 days for vaccinated mice for complete closure of wound [45]. For pregnancy experiments, studies noted decreased fertility in mice immunized with VEGFR2-targeted vaccines [43,45,47]. For example, Nair and colleagues found a moderate effect on fertility with the vaccine in which litter size returned to normal 8 weeks post- vaccination [47]. A more marked effect on fertility was shown with a DC vaccine that had been pulsed with a VEGFR2-alkaline phosphatase fusion protein [45]. The litter size in these mice was decreased to 2 (compared to 6 in the wild-type group) and the mice born were either still born or lived for 10 days. Interestingly, the DC-VEGFR2 fusion protein vaccine had no effect on wound healing.

In contrast to most VEGFR2 vaccines that had an effect on either wound healing and/or fertility, the VEGFR2 DNA vaccine delivered by the Listeria was devoid of delayed wound healing and decreased fertility [32]. Moreover, other angiogenic vaccines including those targeting VEGF-A [47,73], HMW-MAA [33], bFGF [77], TEM8 [83], and survivin vaccines [35] had no effects on wound healing and/or fertility. Although the angiogenic target no doubt plays a role in these side effects (or lack of), the route and delivery vector are likely important contributors to these unwanted side effects. For example, the endothelial DNA vaccines delivered by bacteria were remarkably free of side effects including delayed wound healing and reduced fertility; indeed 4 of the 5 orally delivered bacteria vaccines targeting different angiogenic targets, which examined wound healing and/or fertility, had no side effects [32,33,35,83]; the bacterial vaccine that affected physiologic angiogenesis delayed wound healing minimally with no effect on fertility [45]. Toxicity profiles of many other delivery systems (e.g., electroporation) cannot be fully evaluated because wound healing and fertility studies have not been done.

That anti-angiogenic agents such as Avastin have been well tolerated gives us cautious optimism that other anti-angiogenic strategies such as the use of vaccines may be similarly well-tolerated. Nevertheless, it is clear that the anti-angiogenic agents may have unanticipated side effects beyond their effects on tumor growth, bleeding diathesis, fertility, and wound healing [131]. The side effects of Avastin, thought to be due to its anti-VEGF action, including hypertension, proteinuria, and thrombosis have not been reported for endothelial vaccines [131], but it would not be surprising if some of these side effects occur with VEGF/VEGFR2 targeted vaccines. In summary, the lack of significant toxicity reported by tumor endothelial vaccine pre-clinical studies is encouraging, but circumspection to this vaccine approach is needed based on potential toxicities concerning physiologic angiogenesis and vascular homeostasis.

## Conclusion

Significant resources including numerous pre-clinical and clinical studies have been devoted to the development of tumor vaccines. Thus far, these results have progressed and culminated in the approval a vaccine targeting advanced prostate cancer (Provenge). Although the benefits with this FDA-approved vaccine are modest, further improved vaccine versions are no doubt on the horizon and will aid with other vaccines approaches, including those against the tumor endothelium. Compared to tumor vaccines, the number of varied approaches for tumor endothelial vaccines is relatively limited and is currently restricted to pre-clinical experiments and primarily phase I trials.

As discussed in this review, several studies with tumor endothelial vaccines show anti-tumor efficacy in both transplantable and transgenic tumor-bearing animal models. Of particular note were the bacteria-based vaccines that showed marked anti-tumor response with varied anti-angiogenic genes and few if any side effects. Nevertheless, major obstacles still remain, including identification and validation of specific targets on the tumor endothelium, inhibition of local suppression mechanisms, and boosting anti-tumor immunity through NK cells [132]. Thus far, there have been few data from the endothelial vaccine studies on the various T-cell subtypes, including regulatory T-cells or myeloid derived suppressor cells [25,133,134], and comparison of T-cell subtypes in the tumor and peripheral tissues may be useful in development of more effective vaccines. Other immune cells such as NK cells have not yet shown a direct role in augmenting the efficacy of tumor endothelial vaccines [64,87,94,130], but more research examining interactions among NK cells, dendritic cells, and immunomodulatory agents is needed [132,135,136].

Since tumor angiogenesis is a complex process, targeting a single epitope is unlikely to be successful. In many cases, the treated tumor adapts and finds alternative mechanisms of tumorigenesis eventually leading to resistance to therapy. Thus, combinations of anti-angiogenic vaccines with existing chemotherapy or immunomodulatory therapies offer interesting and exciting possibilities. For example, as discussed previously in this review, combinatory treatments between vaccines and IL-12, GM-CSF, CCL21, or β-defensins markedly increased the immune response toward tumor endothelial cells [39,41,62,64]. Nevertheless, these co-stimulatory therapies have been used sparingly and other commonly used cytokines such as IL-2, IFN α or β [137-139] have not been co-administered or transfected into immune and/or endothelial cells to augment vaccine efficacy. Moreover, considerable more research is needed to determine the optimal co-stimulatory therapy to be administered with the vaccine for the different delivery methods.

Another consideration in developing anti-angiogenic vaccines is their potential for causing complications. Cross-reactivity between tumor and non-tumor disease tissues due to tumor endothelial vaccine may result in reduced compensatory biological processes. The classic tumor endothelial target is VEGFR2 up-regulated not only in the endothelial vessels of tumors but also in healing wounds and hypoxic cardiac tissues. Interestingly, at least with 4 vaccine studies (VEGFR2, VEGF-A, bFGF, survivin/CCL21) tumor angiogenesis was markedly inhibited, but these vaccines did not interfere with normal physiologic processes in several studies [32,35,47,76,77]. The mechanism whereby tolerance to self-angiogenic antigens in tumors but not in normal angiogenic processes is broken remains unknown. It has been suggested that differences in breaking self-angiogenic antigen tolerance between tumors and normal physiological processes may be based on the difference of their vascular organization [32,140]; determining whether or not this is the mechanism for this difference will require further study.

To ensure effective tumor eradication and reduce autoimmune side effects, intensive efforts are still needed to identify additional targets specific to the tumor endothelium. One such study recently found highly specific and expressed markers of the tumor endothelium that were not expressed in quiescent blood vessels or physiologic angiogenesis [13]. Nevertheless, the efficacy of vaccines against these new markers has not yet been determined. Alternatively, finding tissue-specific vascular targets (e.g., prostate) may enable development of tumor endothelial vaccines with acceptable side effects [141,142]. There is also the possibility that tailored endothelial vaccines may be developed based on specific endothelial epitopes associated with certain tumors [143]. As new anti-angiogenic targets are discovered, we anticipate that promising new therapeutic approaches are on the horizon.

## Abbreviations

bFGF: basic fibroblast growth factor; CTL: cytotoxic T-lymphocyte; DC: dendritic cells; FGFR-1: fibroblast growth factor receptor-1; GRP: gastrin-releasing peptide; GM-CSF: granulocyte macrophage-colony stimulating factor; HMW-MAA: High Molecular Weight-Melanoma Associated Antigen; HUVECs: human umbilical vein endothelial cells; IFN: interferon; im: intramuscular; ip: intraperioneal; IL: interleukin; *Lm*-LLO-HMW-MAA: *Listeria monocytogenes *that contains and secretes a fragment of HMW-MAA fused to the N-terminal listeriolysin O; MHC I: Major Histocompatibility Complex I; MMP-2: Matrix Metalloproteinase-2; NK: Natural Killer cells; PDGFR-β: platelet derived growth factor receptor beta; TEM: tumor endothelial marker; Th: T helper; VEGF: vascular endothelial growth factor.

## Competing interests

The authors declare that they have no competing interests.

## Authors' contributions

AM, QL, S-TC, and AJM were involved in preparation of the manuscript. AM and AJM also revised the manuscript for final approval. All authors have read and approved the final manuscript.
